# ONCOGRAM: study protocol for the evaluation of therapeutic response and survival of metastatic colorectal cancer patients treated according to the guidelines of a chemosensitivity assay, the Oncogramme®

**DOI:** 10.1186/s13063-021-05531-y

**Published:** 2021-08-21

**Authors:** Muriel Mathonnet, Mathieu Vanderstraete, Christophe Bounaix Morand du Puch, Stéphanie Giraud, Christophe Lautrette, Mehdi Ouaissi, Nicolas Tabchouri, Abdelkader Taïbi, Renaud Martin, Isabelle Herafa, Achille Tchalla, Niki Christou, The ONCOGRAM trial investigators, B. Marin, S. Bouvier, S. Durand-Fontanier, A. Fabre, D. Valleix, T. Rivaille, F. Fredon, S. Derbal, P. Carrier, R. Daloko Lonfo, R. Legros, S. Lavau-Denes, V. Lebrun-Ly, F. Thuillier, P. Engel, A. Chaunavel, M. Pradel, D. Pezet, A. Dubois, C. Pétorin, O. Antomarchi, A. Aboukassem, A. Vimal-Baguet, B. Gillet, B. Mathieu, J. Joubert-Zakeyh, S. Evrard, Y. Becouarn, D. Béchade, M. Fonk, G. Desolneux, N. Dauriat, M. Agbo, M. Louty, F. Borie, S. Lyubimova, V. Phoutthasang, B. Brunaud-Gagniard, Y. Benadjaoud, N. Rolland, L. Letournoux, P. Roger, L. Chen, Z. Amadou, C. Christopoulous, G. Nakahl, Y. Souliman, M. N. Cirt, D. Ducoux, P. A. Boisseau, P. Pardies, L. Mesturoux, L. Vayre, A. Abdeh, F. Teboul, R. Landraud, M. Ouaissi, E. Salamé, N. Tabchouri, T. Lecomte, G. Proutheau, S. Guyetant, D. Tougeron, A. de Singly, A. Ferru, R. El Fadel, T. Courvoisier, A. Junca, E. Frouin, L. Rouleau, S. Rafaert, A. Rocher, J-M Regimbeau, C. Sabbagh, E. Dumange, E. Chive, D. Lignier, N. Siembida, B. Chauffert, V. Hautefeuille, D. Chatelain, E. Rivkine

**Affiliations:** 1 Department of Digestive, General and Endocrinology Surgery, Dupuytren University Hospital, 2 Avenue Martin Luther King, Limoges, France; 2 EA3842 laboratory (CAPTuR: “Contrôle de l’Activation cellulaire, Progression Tumorale et Résistances thérapeutiques”), Limoges Medical School, 2 rue du docteur Marcland, Limoges, France; 3 Oncomedics SAS, 1 Avenue d’ESTER, Limoges, France; 4 Department of Digestive, Oncological, Endocrine, Hepato-Biliary, Pancreatic and Liver Transplant Surgery, Trousseau University Hospital, Avenue de la République, Chambray-lès-Tours, France; 5 Research and Innovation Bureau, Dupuytren University Hospital, 2 Avenue Martin Luther King, Limoges, France; 6 Centre of Clinical Investigation 1435, Dupuytren University Hospital, 2 Avenue Martin Luther King, Limoges, France; 7 Department of Clinical Geriatrics, Dupuytren University Hospital, Limoges, France

**Keywords:** Colorectal cancer, Oncogramme®, ONCOGRAM, Metastatic, Functional assay, CSRA, Personalized medicine, Chemosensitivity

## Abstract

**Background:**

Colorectal cancer is a major public concern, being the second deadliest cancer in the world. Whereas survival is high for localized forms, metastatic colorectal cancer has showed poor prognosis, with a 5-year survival barely surpassing 11%. Conventional chemotherapies against this disease proved their efficiency and remain essential in first-line treatment. However, the large number of authorized protocols complexifies treatment decision. In common practice, such decision is made on an empirical basis, by assessing benefits and risks for the patient. In other words, there is currently no efficient means of predicting the efficacy of any chemotherapy protocol for metastatic colorectal cancer.

**Methods/design:**

The use of a chemosensitivity assay, the Oncogramme®, should help clinicians administer the best chemotherapy regimen to their patients. We hypothesize it would ultimately improve their survival. In this multicentred, prospective trial (ONCOGRAM), eligible patients with metastatic colorectal cancer are randomized to determine whether they will receive an Oncogramme®. For clinicians whose patients benefited from the assay (arm A), results are used as a decision support tool. Patients not undergoing the Oncogramme® procedure are treated according to current practice, without the assistance of the assay (arm B). Primary outcome is 1-year progression-free survival. Secondary outcomes include response rates, as well as 6-month and 1-year survival rates.

**Discussion:**

This study aims at investigating the clinical utility of the Oncogramme® as a decision support tool for the treatment of patients with metastatic colorectal cancer. If the Oncogramme® positively influenced patient overall survival and/or progression-free survival, it would be of great value for clinicians to implement this assay within the current landscape of personalized medicine tools, which include genomics and biomarker assays.

**Trial registration:**

ClinicalTrials.gov identifier NCT03133273. Registered on April 28, 2017.

## Background

Colorectal cancer (CRC) is a major public health concern, being the second leading cause of cancer-related mortality worldwide, with about 880,000 deaths each year [1]. CRC is tightly correlated to high human development index (HDI), with a higher incidence in Western Europe, North America, and Oceania. As a result, rises of both incidence and mortality were recently observed in countries considered as emerging such as China and Brazil [2]. Worldwide, however, a decreased overall mortality has been observed over the last three decades. This is mainly due to earlier and more effective detection methods, as well as better treatments [3]. This encouraging trend is obscured by the poor survival rate of patients with metastatic CRC (mCRC), which is around 11.4% [4]. Efforts are made to improve survival by developing more efficient treatments, but also personalized medicine tools to better evaluate patient tumour’s phenotypic and genomic characteristics. For mCRC treatment, a wide panel of drug combinations are available. Selection of chemotherapy regimens relies on empirical decisions, balancing potential benefits and toxicity [5, 6]. Current standards of care are combinations of 5-fluorouracile (5-FU) and folinic acid (FA, also known as leucovorin) with either oxaliplatin (FOLFOX), irinotecan (FOLFIRI), or both (FOLFIRINOX and FOLFOXIRI). These combinations may be supplemented with anti-VEGF (bevacizumab) or anti-EGFR (cetuximab or panitumumab) antibodies, depending on the tumour’s *KRAS*/*NRAS/BRAF* mutational status [7]. Relapsing, microsatellite instable (MSI) mCRC patients may also receive immune checkpoint inhibitors nivolumab or pembrolizumab [8, 9]. When used as first-line treatment, doublet regimen FOLFIRI and FOLFOX show similar response rates and OS profiles, yet the latter’s administration frequency is much higher and continuously increasing [10]. Also, in first-line setting, FOLFIRINOX performs better than other combinations [11]. However, it induces more severe side effects, which may lead to empirical dosing reduction or treatment discontinuation [12]; it is thus usually limited to more robust patients. In any case, there is currently no data available to indicate whether appropriately targeted untreated or relapsing patient subpopulations would benefit even more from either drug combination.

To assist clinicians in their decision-making process, chemosensitivity and chemoresistance assays (CSRA), as members of the larger family of functional assays, have been developed against a wide variety of cancers. They aim at predicting chemo-response based on ex vivo culture of a patient’s own tumour sample. Numerous CSRA have been developed in the last three decades, reaching high technical and clinical accuracies [13]. Despite these encouraging results, CSRA are still considered as investigational, as underlined by the latest clinical practice guidelines published by the American Society of Clinical Oncology (ASCO) [14]. These recommendations result from insufficient clinical evidence. However, given the potential value of functional assays, the ASCO strongly encourages the implementation of randomized, controlled, prospective trials [15, 16].

The Oncogramme®, a CE-marked in vitro diagnostics medical device (IVD-MD) developed by the French company Oncomedics, is dedicated to cancer treatment decision support [17–19]. Briefly, it consists in the measurement of therapy-induced mortality on patient sample primary cultures using fluorescence microscopy. This test is fully standardized, allowing both reliability and a high success rate. When applied to mCRC, the Oncogramme® directly evaluates drug combinations commonly used by oncologists, such as 5-FU, FOLFOX, FOLFIRI, or FOLFIRINOX. A pilot study performed on a cohort of patients with mCRC showed a sensitivity of 84.6% of the assay in predicting tumour response [20], which is sensibly higher to published literature on CRC chemosensitivity assays [21].

To our knowledge, most clinical studies involving functional assays are retrospective studies [13]. There is a pressing need for information about the utility of these assays in actively assisting clinicians in making a therapeutic decision [14]. The ONCOGRAM trial is a multicentric, randomized, two-arm, single-blind, prospective and interventional study that will give insights into the clinical value of the Oncogramme®, as well as into its potential as a trustable and reliable tool for treatment individualization by oncologists.

## Methods/design

### Objectives

The primary outcome for this study is progression-free survival (PFS) 1 year following inclusion.

Secondary outcomes include the following:

- Response rate;
- 6-month and 1-year overall survival (OS);
- 6-month PFS;
- Incremental cost-effectiveness ratio (ICER): expressed in euros per year of life gained without occurrence of death or progression to 1 year of the adapted chemotherapeutic treatment according to the results of the Oncogramme® using the EQ5D-5 L scale;
- Incremental cost/utility ratio (ICUR): expressed in euros per quality-adjusted life year (QALY) gained at 1 year of the adapted chemotherapeutic treatment according to the results of the Oncogramme® using the EQ5D-5 L scale;
- Reasons for which clinicians did not follow results of the Oncogramme®;
- Proportions of grade 3 to 5 adverse events (AE) according to the following classification: grade 1, mild AE; grade 2, moderate AE; grade 3, severe AE; grade 4, life-threatening or disabling AE; grade 5, death related to AE.

The occurrence of an adverse event is reported immediately to the sponsor. Pregnancy is not an adverse event but is reported to the sponsor.

### Study population and design

This is a randomized, two-arm, single-blind, prospective, interventional study, involving 13 French clinical centres with a competitive enrolment. Eligible patients must be at least 18 and have a pre-operatively or per-operatively diagnosed metastatic colon or rectal cancer. Staging must be confirmed histologically, and metastases must be measurable according to *response evaluation criteria in solid tumours* (RECIST 1.1) [22]. Patients must be able to be treated using a standard chemotherapy regimen, including 5-FU-based therapies FOLFOX, FOLFIRI and FOLFIRINOX (or FOLFOXIRI). Chemotherapies may be associated with targeted therapies such as anti-angiogenic antibodies (bevacizumab), and anti-EGFR antibodies (panitumumab and cetuximab), suitable for patients with wild type *BRAF*/*NRAS*/*KRAS* tumours. Chemotherapy must have a curative or palliative aim.

Patients fulfilling any of the following criteria are excluded from the trial:

- Contraindication to paraclinical exploration;
- Contraindication to any chemotherapy treatment;
- Exclusive use of radiotherapy, targeted therapy or immunotherapy, or exclusive palliative support care;
- Women of childbearing age who do not use contraception; pregnant and breastfeeding women;
- Patients with a legal guardian; patients who are not capable of understanding the terms of the trial.

**Fig. 1:**
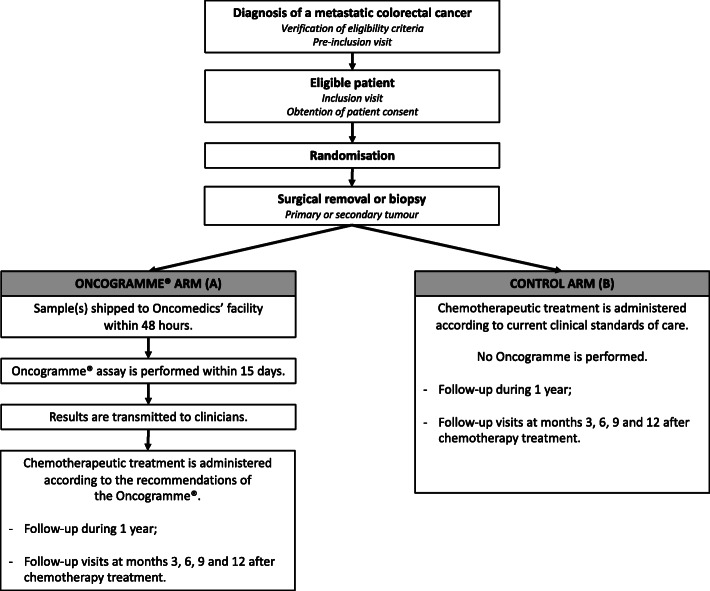
ONCOGRAM diagram for confirmed mCRC samples. When mCRC is already histologically diagnosed, inclusion and randomization occur before performing the Oncogramme®. Oncomedics only receives samples and realizes the assays for patients already randomized in the Oncogramme® arm

**Fig. 2:**
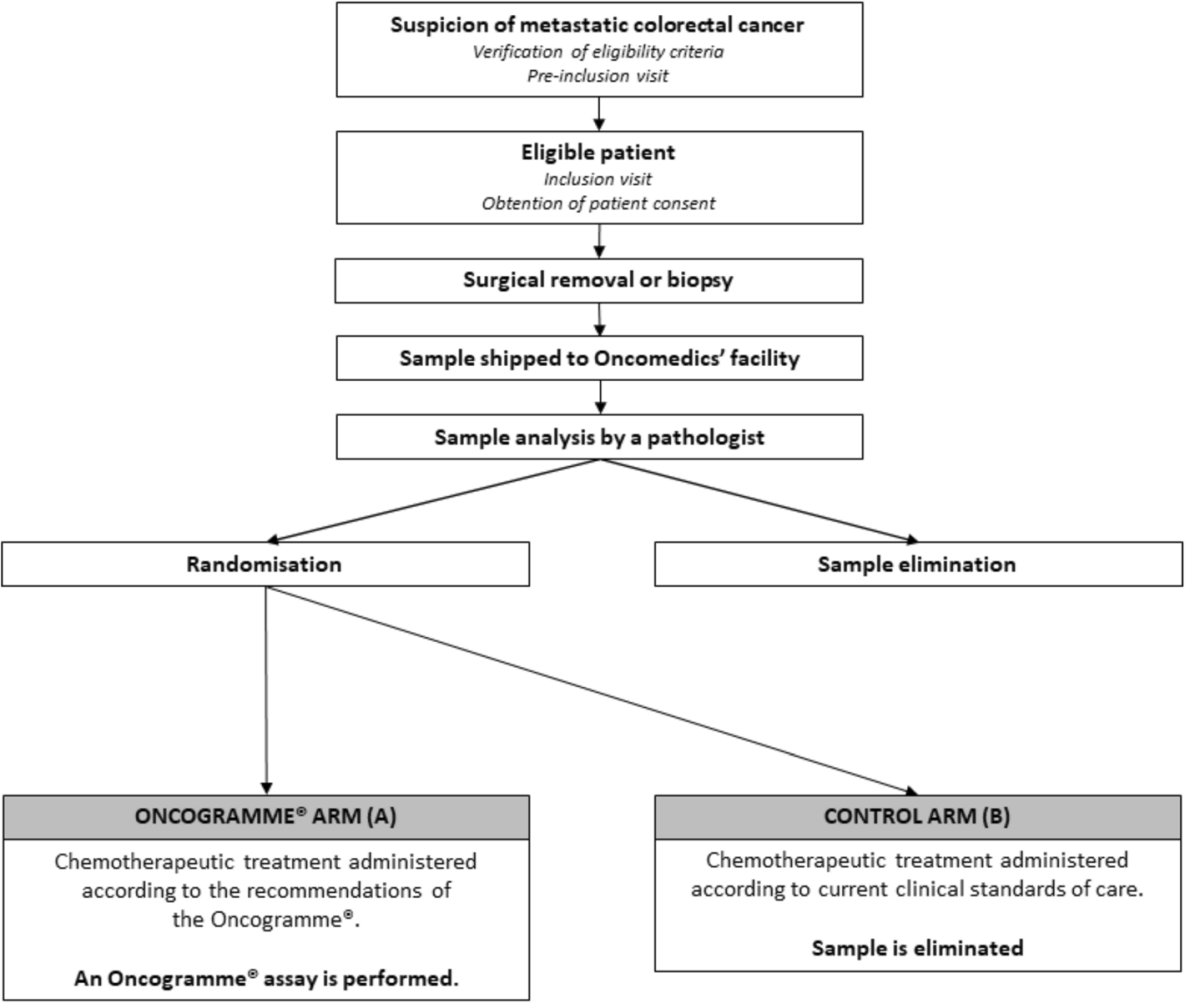
ONCOGRAM diagram for suspected mCRC samples. Oncomedics receives and starts the Oncogramme® procedure on all samples not histologically qualified yet. Randomization occurs if the sample is confirmed as mCRC. Realization of the Oncogramme® is pursued only if the patient is randomized in the Oncogramme® arm. In all other cases, the sample is eliminated

The study follows an intention-to-treat scheme: this means the clinical response of a patient will be analysed according to the group to which the patient was assigned through randomization, independently of the fact that the Oncogramme® chemosensitivity profile was actually utilized or not to treat this patient (Fig. 1). A slightly different scheme has been designed to allow suspected mCRC samples to be integrated into the study (Fig. 2).

Patients receive comprehensive information about the research prior to signing an informed consent form (ICF). They are subsequently randomized by the clinical centre where they receive treatment through a secured internet randomization platform. This platform is handled by the Limoges University Hospital biostatistics department (*Centre d'Épidémiologie, de Biostatistique, et de Méthodologie de la Recherche*, CEBIMER). The randomization software automatically determines the arm and provides clinical research associates and Oncomedics’ personnel relevant information about each subject: patient’s identification number, age and year of birth, sex, arm, name of clinician responsible for the randomization, clinical centre’s name, date of ICF signature. Patient randomization is performed using the modules *CSOnline* and *CSRandomization* of the software *Ennov Clinical* v8.0.120 (Ennov, Floirac, France). Patients are allocated to each arm by the method of minimization, using age, gender and centre as stratification factors. All patient data obtained for or generated by the trial are recorded in case report forms, whose information is incorporated into a dedicated, secured database generated using *Ennov Clinical* and its *CSDesigner* module. A participant may withdraw or withdraw consent to participate in the research at any time. In this case, he/she will no longer be followed in the protocol but will continue to receive the best possible care.

Following randomization, follow-up visits occur on a regular basis: first before chemotherapy treatment, then after 3, 6, 9 and 12 months. EQ5D-5 L questionnaires are filled by patients after every post-chemotherapy visit (Table 1). Total study duration for every patient is 12 months.

**Table 1:** Overview of patient management steps during the whole course of the ONCOGRAM trial

Patient management step	Pre-inclusion day − 30 to day − 1	Inclusion day 0	Before first chemotherapy treatment	Follow-up visits	Final follow-up visit day 0 + 12 months
Information given to patient	**✓**				
Check eligibility criteria	**✓**	**✓**			
Obtain patient's informed consent		**✓**			
Randomization		**✓**			
Clinical examination^a^		**✓**		**✓**	**✓**
Biological examination	**✓**^b^	**✓**^b^		**✓**^b^	**✓**^b^
Paraclinical examination^c^		**✓**		**✓**	**✓**
EQ5D-5 L questionnaires		**✓**^d^	**✓**^d^	**✓**^d^	**✓**^d^
Search for adverse effects of chemotherapies				**✓**	**✓**

^a^Clinical examination: weight, height, cardiopulmonary checkup, comorbidities assessment, WHO performance status

^b^Albumin, protides, CEA, CA 19-9, complete blood count, liver function test (creatinine, AST, ALT, alkaline phosphatase, bilirubin, GGT): compulsory for inclusion and at months 1, 3, 6, 9 and 12. A urinary/blood pregnancy test is also performed for women of procreating age before inclusion

^c^Paraclinical examination includes if needed colonoscopy, MRI, PET scan, abdominal ultrasonography, thoraco-abdominopelvic CT

^d^EQ5D-5 L questionnaires are filled by the patient before first chemotherapy, then at months 3, 6, 9 and 12

If a patient withdraws their consent, all data will be removed from the database and will not be used for analysis. In case of study discontinuation, corresponding data might be kept if the patient authorizes it. In that context, primary and secondary outcomes will be evaluated at the date of discontinuation.

The membership of the associated centres is maintained through monthly newsletters where the rate of inclusion is reported for each centre. A blog has been created. Individual webinars are held with all centres twice a year.

### Sample size calculation

Based on a 1-year PFS of 15% in the control arm, and 30% in the Oncogramme®-assisted arm, a log-rank test evaluated the total number of patients to include at 204 (with a significance level of 0.05 and a power of 80%). Sample size ratio is 1:1, meaning that each arm should include 102 patients. Taking into consideration the eventuality of non-assessable patients, 20% has been added to the initial sample size, which eventually reached 256. Based on clinical data observed at Limoges hospital, it has been estimated that the study should include 5 patients per month, allowing to reach the target population of 256 patients in a 48-month timeframe.

### Statistical analysis

Intent-to-treat analyses will be performed by the CEBIMER with the use of SAS® 9.3 software. Results will be reported according to the CONSORT 2010 Statement [23].

1-year PFS will be measured in each arm using the Kaplan-Meier method, with 95% confidence intervals. Then, comparison between the two groups will be performed using a log-rank test. Starting point for PFS measurement is the randomization date. Ending points are (*i*) event date for patients showing evidence of progression, (*ii*) date of last visit for lost to follow-up patients, and (*iii*) data cut-off date for living patients with no progression.

Secondary analyses will be performed as follows:

- Response rate: comparisons between response rates in control and Oncogramme® arms will be performed using a Chi^2^ or a Fisher’s exact test, depending on whether conditions of application of Chi^2^ are met;
- 6-month and 1-year OS: values will be estimated similarly as the primary outcome, using a Kaplan-Meier test with 95% confidence intervals;
- Disease-free survival: Kaplan-Meier test with 95% confidence intervals;
- Incremental cost-effectiveness ratio: ICER will be expressed as euros per QALY for patients who benefited from the Oncogramme®;
- Comparison of EQ5D-5 L utility scores: comparison will be performed using a *t* test or a non-parametric Mann-Whitney test, depending on whether conditions of application are met.
- Comparison of the proportions of grade 3 to 5 adverse events: Chi^2^ or a Fisher’s exact test, depending on whether conditions of application of Chi^2^ are met.

### Procedure of the Oncogramme®

#### Sample preparation and shipment

Following exeresis, a fresh specimen containing tumour tissue is qualified by pathologists, then shipped to Oncomedics’ laboratory according to UN3373 classification standards. The use of a dedicated transportation medium (OncoMiD-Via for colon, Oncomedics) allows preservation of sample quality and viability for further use. The ideal timeframe between patient surgery and sample processing should not exceed 48 h to ensure sample optimal quality. Only samples for patients assigned to arm A are sent to Oncomedics.

#### Sample processing

After decontamination, the tumour sample is dissociated using a proprietary method combining both mechanical and enzymatic steps [17, 19, 20]. Following dissociation, cell viability is measured by trypan blue exclusion assay (Merck). Then, the cell suspension is cultivated in a CRC-specific, defined (serum-free) medium (OncoMiD for colon, Oncomedics). Cells are incubated at 37 °C under a 5% CO_2_ atmosphere for 6–8 days, with medium renewal after 4 to 5 days. This step allows progressive elimination of non-tumoral cells to retrieve a majority of epithelial cells.

#### Chemotherapy treatment

Tumour cells are seeded in 8-well LabTek chamber slides (Thermo) at a concentration of 10^5^ cells per mL. For a given cell culture, five conditions are prepared in triplicate: untreated; 5-FU + FA; FOLFIRI (5-FU + AF + irinotecan); FOLFOX (5-FU +AF + oxaliplatin); FOLFIRINOX (5-FU + AF + irinotecan + oxaliplatin) (all chemotherapies provided by Merck). Critical chemotherapy concentrations were determined as previously described [17, 19, 20]. Cells are then incubated for 72 h at 37 °C under a 5% CO_2_ atmosphere before labelling.

#### Labelling and mounting

Following exposure to treatments, cell viability is assessed through a fluorescent triple labelling. Briefly, cells are incubated for 45 min in PBS containing 4 μM acetomethoxy derivate of calcein and 0.1 μM ethidium homodimer-1 (LIVE/DEAD® viability/cytotoxicity kit, Life Technologies). Cells are then fixed at 22 °C for 30 min using 4% formaldehyde in PBS (Merck). Subsequently, cell nuclei are stained with DAPI (Merck) at 22 °C for 20 min. Slides are dried before mounting with glycerol-gelatin mounting medium (Merck). Finally, slides are stored at − 20 °C until readout.

#### Readout and analysis

Analysis is performed using a fluorescent upright microscope (Eclipse 80i, Nikon). For each well, several multi-channel fields are pictured over the whole surface using NIS-Elements BR 3.1 software (Nikon). Variable number of pictures are taken for each patient, to provide a sufficient number of cells and obtain statistically robust data. Cell mortality is then manually assessed based on fluorescence profiles. Sensitivity thresholds determined following a previous study (except for FOLFIRINOX) [20] are applied to cell death measurements to determine the sample’s chemosensitivity to a particular drug combination. These thresholds correspond to cell mortality ratio values for which a tumour is considered as sensitive to a treatment : hence, if the ratio “cell mortality _*treatment x*_ */* cell mortality _*untreated*_” is superior to “threshold _*treatment x*_”, then the tumour is considered to be sensitive to the treatment. Otherwise, the tumour is considered to be non-sensitive.

### Use of the Oncogramme® results by clinicians

Results of the Oncogramme® are transmitted to clinicians in charge of the patient within 15 working days. This short time frame allows clinicians to use results of the Oncogramme® during multidisciplinary meetings determining the best treatment for each patient. It is worth noting that results of the Oncogramme®, similar to other complementary diagnostics, represent a supplementary tool: the ultimate choice of a patient’s treatment always proceeds from clinicians’ decision. Noteworthy, clinicians must utilize the Oncogramme® results only as an index of chemosensitivity. In this context, negative chemosensitivity results should not be interpreted as chemoresistance.

## Discussion

Precision cancer medicine is a rapidly evolving concept that brings tools to help tailoring patient treatments. The implementation of such tests in common practice necessitates a clinical evidence-based framework to ensure their added value. Hence, as recommended by the ASCO [14], large randomized controlled clinical trials are needed to demonstrate the utility of companion diagnostics and functional assays such as CSRA. Available clinical evidence for functional assays is mostly retrospective and does not study the role of the assay in decision making. This clinical trial aims at bringing insights into the proactive role of such assays in facilitating clinicians’ decisions. We hypothesize the use of the Oncogramme® will improve the percentage of mCRC patients reaching 1-year PFS from 15% to 30%. We assume this would lead to a range of benefits for patients, including better quality of life, less adverse effects and, ultimately, OS improvement. Also, from a medico-economic standpoint, one can assume that choosing the right chemotherapy as soon as first-line regimens would diminish global costs of patients’ care, notably by avoiding the administration of non-effective drugs.

Taken together, positive outcomes of the ONCOGRAM trial would strongly encourage the development of other Oncogramme®-based clinical studies on other malignant pathologies. Preclinical studies have already demonstrated the feasibility of the procedure with ovary and breast tumour samples [18, 19, 24].

## Trial status

The current version of the protocol in use is n° 6.0 (June 19, 2019). The first patient was enrolled on July 24, 2017. Recruitment is expected to end in July 2021. So far, 118 patients out of the 256 initially planned have been enrolled.

## Data Availability

The access to the protocol is possible with Clinical Trials (ClinicalTrials.gov). The participant-level dataset and statistical code will be accessible at the end of the inclusions.

## Abbreviations

CRC: Colorectal cancer
mCRC: Metastatic colorectal cancer
CSRA: Chemosensitivity and resistance assay
PFS: Progression-free survival
OS: Overall survival
FA: Folinic acid
FOLFOX: FA + leucovorin + oxaliplatin
FOLFIRI: FA + leucovorin + irinotecan
FOLFIRINOX: FA + leucovorin + oxaliplatin + irinotecan
IVD-MD: In vitro diagnostics medical device
RECIST: Response evaluation criteria in solid tumours
